# A Case of Gillespie Syndrome With Atypical Presentation

**DOI:** 10.7759/cureus.31341

**Published:** 2022-11-10

**Authors:** Gurdeep Singh, Saketh Narahari

**Affiliations:** 1 Endocrinology, Diabetes and Metabolism, Our Lady of Lourdes Memorial Hospital, Binghamton, USA; 2 Internal Medicine, Lake Erie College of Osteopathic Medicine, Bradenton, USA

**Keywords:** itpr1, cerebellar ataxia, aniridia, inherited disorders, gillespie syndrome

## Abstract

Gillespie syndrome, a genetically inherited condition, is described as a disease that primarily affects the ocular and associated nervous systems. It is characterized by a clinical triad of bilateral aniridia, intellectual disability, and cerebellar ataxia, and is inherited in an autosomal dominant or recessive fashion. The most well-studied mutations related to this syndrome affect the inositol 1,4,5-trisphosphate receptor type 1 gene (ITPR1). Gillespie syndrome is an exceptionally uncommon diagnosis with less than 50 patients ever being diagnosed. We present a case of a patient with bilateral aniridia and ataxia but lacking intellectual disability, and moreover had no known family history of this syndrome. Our case report shows that Gillespie syndrome may not necessarily present with the classic “triad” of symptoms as previously described in the literature.

## Introduction

Gillespie syndrome is an ultra-rare genetic disease classically known as a triad of bilateral aniridia, intellectual disability, and cerebellar ataxia [1]. It can be inherited in either an autosomal dominant or autosomal recessive manner, and the most common mutations usually affect the inositol 1,4,5-trisphosphate receptor type 1 gene (ITPR1) [2]. IPPR1 encodes for a Ca2+ channel receptor on the endoplasmic reticulum. Recent research suggests that the ITPR1 receptor is involved in the differentiation of anterior eye segment tissues [3].

Gillespie syndrome is characterized by a constellation of findings that affect multiple body systems and is usually diagnosed in early childhood, where eye examinations reveal bilateral aniridia [4]. Aniridia can be confirmed on slit-lamp examination, which may further reveal scalloped pupillary margins, which are pathognomonic of Gillespie syndrome when associated with aniridia. Other early signs of Gillepsie syndrome include hypotonia and mental retardation, which in the setting of aniridia raises concern for Gillespie syndrome. Mental retardation is another early presenting sign which manifests as delayed developmental milestones. CT and MRI scans in childhood show cerebellar hypoplasia, in many cases before the age of 10 years. Such findings help confirm the diagnosis. In this article, we present a unique case of a patient with Gillespie syndrome who presented with bilateral aniridia, mild to no intellectual disability, and non-progressive cerebellar ataxia.

## Case presentation

**Figure 1:**
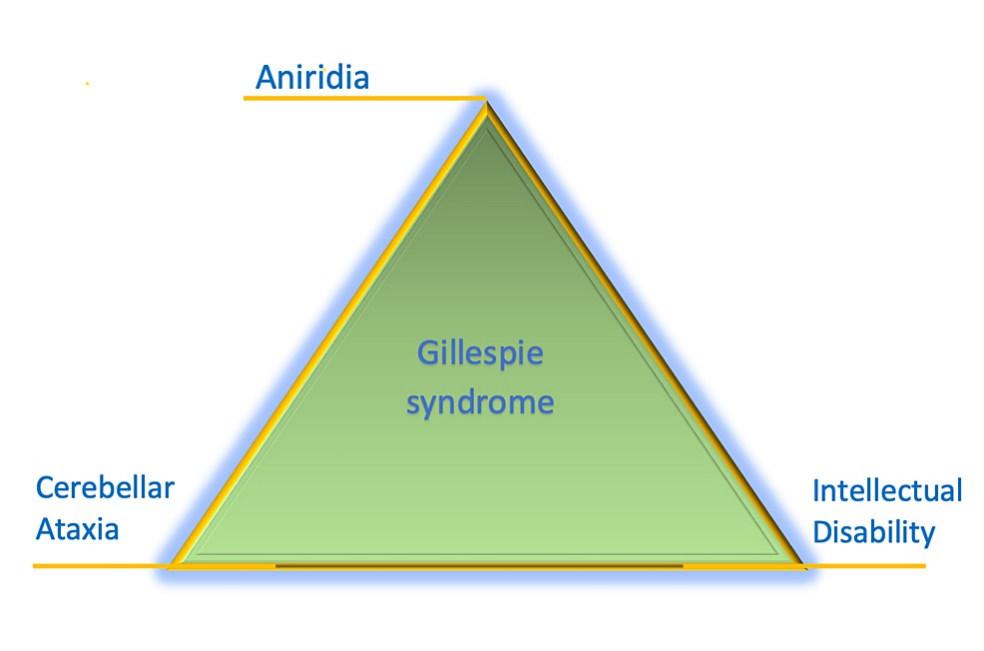
The Classic Triad of Gillespie Syndrome

The patient was a 73-year-old Caucasian male who initially presented to the emergency room with left-sided numbness with an ataxic gait. The patient was diagnosed with aniridia by an ophthalmologist during childhood. He had a long history of poor balance with an abnormal gait. He also reported that he is slow to respond to commands. The neurological examination revealed axial-locomotor ataxia at bilateral lower extremities, and mild dysmetria on the finger-to-nose maneuver bilaterally. The ophthalmologic examination showed scalloped pupils that were dilated and fixed bilaterally, pupils were non-reactive to light, which is a typical nonspecific examination finding in patients with Gillespie syndrome [5-6]. The exam also revealed pseudophakia, consistent with his past history of bilateral cataracts which were removed by surgery. Visual acuity was also decreased, and the patient reported blurry vision. Mental status examination revealed a normal-appearing individual with a cooperative effect. The patient was making appropriate eye contact. He was alert and oriented to person, place, and time. Mini Mental State Exam (MMSE) showed a score of 27 with normal memory and attention. This is inconsistent with the classic triad [1], which includes intellectual disability (Figure 1). Language was fluent with no aphasia. His thoughts were coherent. His siblings and parents were unaffected by ophthalmic and neurological syndromes, and there was no evidence of Gillespie syndrome in the family. 

**Figure 2:**
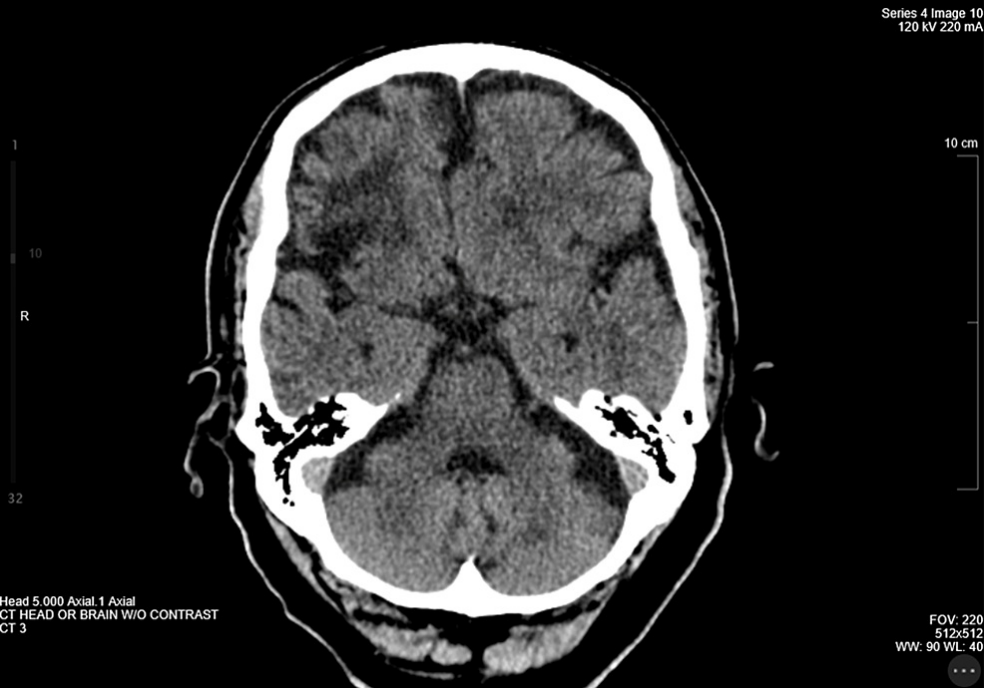
Posterior Fossa CT Scan Findings

A CT brain without contrast showed cerebral cortical atrophy, specifically frontotemporal. CT scans also revealed mild white matter microvascular changes and cerebral volume loss. Visualization of the posterior fossa revealed an unremarkable cerebellum and brainstem (Figure 2). 

**Figure 3:**
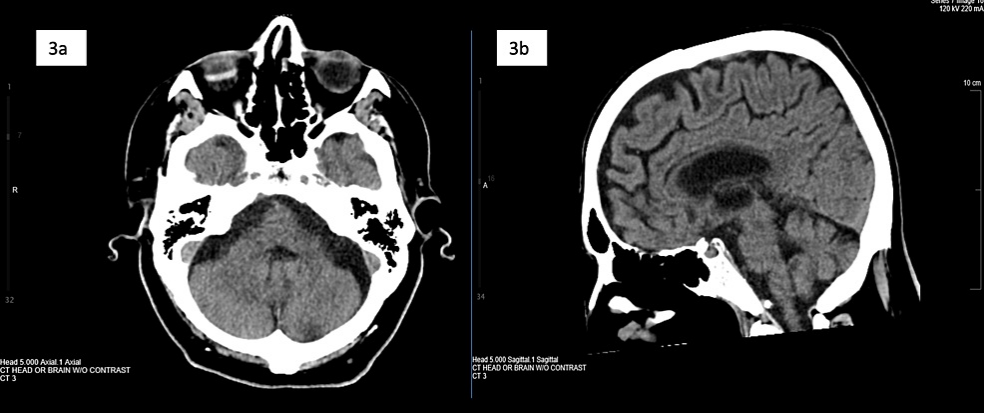
Repeat CT Scan Findings Showing Cerebellum and Brainstem, Axial and Sagittal Views

Repeat brain CT showed encephalomalacia consistent with prior infarcts in the thalamus with no posterior fossa findings (Figures 3a-3b).

**Figure 4:**
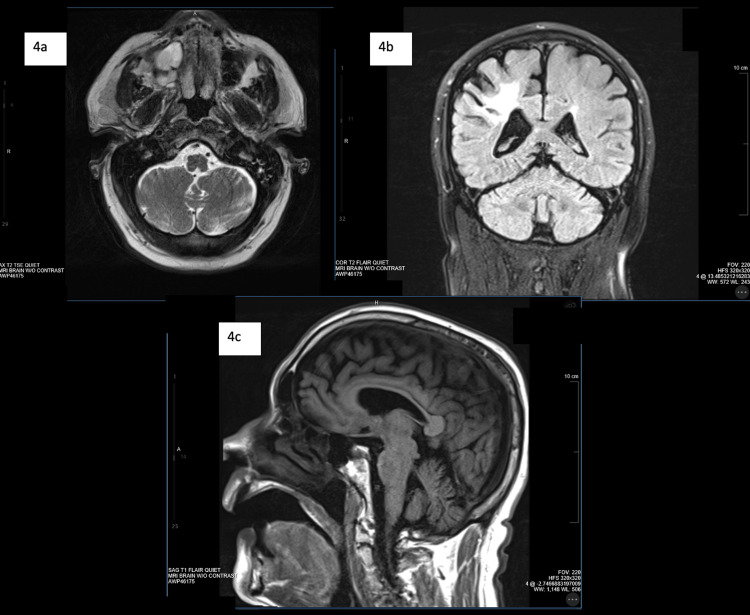
MRI Non-contrast Findings

MRI scans confirmed these findings, which showed volume loss in the body of the corpus callosum suggestive of moderate cerebral atrophy but no posterior fossa findings (Figures 4a-4c). Interestingly, the typical finding of cerebellar vermis atrophy was not found in any of the CT and MRI scans.

## Discussion

Gillespie syndrome is a rare genetic condition primarily affecting the ophthalmic and neurologic systems (Table 1). Aniridia can be an isolated condition or can be a symptom of a more complex disease, such as Wilms tumor, aniridia, genitourinary anomalies, and mental retardation (WAGR) syndrome or Gillespie syndrome [7]. 

**Table 1:** Organ Systems Affected and Associated Symptoms The most common symptoms in each organ system are indicated in bold letters.

System	Symptoms
CNS	Cerebellar Ataxia, hypotonia, hearing loss, cerebral hypoplasia
Ophthalmic	Aniridia, Scalloping of pupillary edges, Mydriasis, nystagmus
Psychosocial	Intellectual Disability, delayed milestones in children
Other	Mask like facies

In this study, we present a rather distinctive case of Gillespie syndrome, with the patient having mild cerebellar ataxia with no corresponding cerebellar changes on CT scan or MRI. Patients with Gillespie syndrome often have noticeable cerebellar vermis atrophy on imaging, along with diffuse cerebral lobe hypoplasia and nonspecific white matter changes [8]. Per our knowledge, this is the first Gillespie syndrome case where cerebellar vermis atrophy on imaging was not a prominent feature and yet neurological examination revealed ataxia. This could potentially signify an error in the radiological readings or can indicate ataxia due to an extra-cerebellar cause. It is also plausible that the ataxia seen in our patient was due to a past thalamic stroke. In the study by Nelson et al., a case was reported where a patient with Gillespie syndrome had a normal CT scan with no specific findings for Gillespie syndrome [1]. In yet another case, a CT scan showed enlargement of the skull base cisterns but otherwise no cerebellar vermal atrophy [9]. The presence of ataxia on physical exam, however, suggested that our patient more likely presented with Gillespie syndrome rather than a case of simple congenital aniridia [10].

The patient also seemed to have little to no intellectual disability. It has been previously argued that intellectual disability should not be a defining feature of Gillespie syndrome. Mariën et al. describe a wide range of behavioral symptoms in previous Gillespie syndrome cases, ranging from dysarthria to severe early-onset mental retardation [11]. In the case report by Stendel et al., the patient with a positive ITPR1 mutation and cerebellar ataxia displayed no cognitive development issues growing up and no intellectual disability [4].

In contrast to the varying presence of cerebellar imaging and intellectual disability, all cases of Gillespie syndrome are known to have aniridia. Aniridia is known to predispose patients to cataracts (50%-85% of patients develop it [10]), and this patient had a history of bilateral cataracts and cataract-removal surgery.

## Conclusions

Less than 50 cases of Gillespie syndrome have been reported since the syndrome was first described. Previous literature suggests that most patients present with some degree of aniridia, intellectual disability, and cerebellar ataxia. With this case report, however, we conclude that intellectual impairment may not be a defining clinical feature of Gillespie syndrome. We highlighted the wide constellation of characteristics that Gillespie syndrome can potentially present with and showed that the classic symptoms may not always be present. 

## References

[1] Nelson J, Flaherty M, Grattan-Smith P (1997). Gillespie syndrome: a report of two further cases. Am J Med Genet.

[2] Gerber S, Alzayady KJ, Burglen L (2016). Recessive and dominant de novo ITPR1 mutations cause Gillespie syndrome. Am J Hum Genet.

[3] Kinoshita A, Ohyama K, Tanimura S (2021). Itpr1 regulates the formation of anterior eye segment tissues derived from neural crest cells. Development.

[4] Stendel C, Wagner M, Rudolph G, Klopstock T (2019). Gillespie's syndrome with minor cerebellar involvement and no intellectual disability associated with a novel ITPR1 mutation: report of a case and literature review. Neuropediatrics.

[5] Saini AG, Sankhyan N, Gupta P, Vyas S, Singhi P (2016). The triad of non-progressive cerebellar ataxia, partial aniridia and psychomotor delay - Gillespie syndrome. Indian J Pediatr.

[6] Nabih O, Hamdani H, ELaaloum L, Allali B, ELkettani A (2022). Gillespie syndrome: an atypical form and review of the literature. Ann Med Surg (Lond).

[7] Hingorani M, Hanson I, van Heyningen V (2012). Aniridia. Eur J Hum Genet.

[8] De Silva D, Williamson KA, Dayasiri KC (2018). Gillespie syndrome in a South Asian child: a case report with confirmation of a heterozygous mutation of the ITPR1 gene and review of the clinical and molecular features. BMC Pediatr.

[9] François J, Lentini F, de Rouck F (1984). Gillespie's syndrome (incomplete aniridia, cerebellar ataxia and oligophrenia). Ophthalmic Paediatr Genet.

[10] Tripathy K, Salini B (2022). Aniridia. Aniridia.

[11] Mariën P, Brouns R, Engelborghs S, Wackenier P, Verhoeven J, Ceulemans B, De Deyn PP (2008). Cerebellar cognitive affective syndrome without global mental retardation in two relatives with Gillespie syndrome. Cortex.

